# Mitigation of Corrosion Initiated by Cl^−^ and SO_4_^2−^-ions in Blast Furnace Cement Concrete Mixed with Sea Water

**DOI:** 10.3390/ma15093003

**Published:** 2022-04-20

**Authors:** Pavel Krivenko, Igor Rudenko, Oleksandr Konstantynovskyi, Danutė Vaičiukynienė

**Affiliations:** 1 Scientific Research Institute for Binders and Materials, Kyiv National University of Construction and Architecture, Povitroflotskyi Prospect 31, 03037 Kyiv, Ukraine; pavlo.kryvenko@gmail.com (P.K.); igor.i.rudenko@gmail.com (I.R.); alexandrkp@gmail.com (O.K.); 2 Faculty of Civil Engineering and Architecture, Kaunas University of Technology, Studentu St. 48, LT-51367 Kaunas, Lithuania

**Keywords:** blast furnace cement, corrosion, minor additional constituent, sea water

## Abstract

The use of blast furnace cement is an effective way to meet the requirements of sustainable development. However, CEM III/C is characterized by slow strength gain. The problem can be worse for plasticized reinforced blast furnace cement concretes mixed with sea water in view of shorter durability. The mitigation of corrosion in plasticized blast furnace cement concretes mixed with sea water can be provided through a composition of minor additional constituents, with percentage by mass of the main constituents: alkali metal compounds, 2…3; calcium aluminate cement, 1; clinoptilolite, 1. The alkali metal compounds are known to activate hydraulic properties of ground granulated blast furnace slag. A calcium aluminate cement promotes the accelerated chemical binding of Cl^−^ and SO_4_^2−^-ions with the formation of Kuzel’s salt. A clinoptilolite occludes these aggressive ions. The positive effects of the mentioned minor additional constituents in the blast furnace cement were supported by the increased early strength gain and the higher structural density, as well as by a good state of steel reinforcement, in the plasticized concretes mixed with sea water.

## 1. Introduction

Current trends in the construction industry predetermine the use of so-called “green” materials in resource- and energy-saving technologies [1], thus reflecting a responsible attitude towards the environment [2]. As a result of a high environmental impact of concrete production and over-exploitation of natural resources, considerable pressure is continuously being exerted on the cement industry to find alternative solutions in order to eliminate use of resources, save energy and limit greenhouse gas emissions [3].

### 1.1. Factors Affecting the Durability of Concretes

One of the most commonly used and widely accepted ways to improve the durability of concretes is the application of so-called blended cements, obtained by a partial replacement of Portland cement clinker by industrial by-products [4]. These by-products include a ground granulated blast furnace slag (further, GGBFS) and fly ash [5], silica fume, natural pozzolans and fillers like trass or limestone [6]. Although the use of various types of blended cements has been encouraged as a green solution, these cements suffer from slow hydration kinetics, resulting in a slower early strength gain, which is a drawback in many construction applications. In order to significantly accelerate the cement hydration process, a number of different methods have been proposed, including the incorporation of nanomaterials, such as, for example, nanosilica [7]. Various studies have confirmed the accelerating action of nanosilica in Portland cement [8] and blended cements [9].

The most known ways to activate latent hydraulic properties of the GGBFS is to apply calcium [10] and sulfate activators [11]. It is also known that low alkaline [12] and nearly neutral salts [13] can be used as activators of blast furnace cements as well. Na (K) salts of strong acids also ensure the increased hydraulic activity of the GGBFS [14]. However, insufficient strength of blast furnace cements and the possibility to only increase early strength are disadvantages of these means of activation: near-neutral salts [15], sulfate [16], etc. The application of oxides or salts of alkaline metals, which provide an alkaline reaction in water, can be a solution to the above-mentioned problem [17]. The increase of the GGBFS content in cement to substitute Portland cement clinker without a loss of strength is possible to achieve in such a way. Alkali activation of aluminosilicate raw materials is now widely used [18].

Concretes based on the GGBFS activated by alkali metal compounds are characterized by the increased strength gain, both early [19] and at 28 d [20], as well as good heat resistance [21], resistance corrosion [22], freeze–thaw resistance [23,24], high waterproofness [25], fire resistance [26,27], etc.

A promising direction being taken to improve the sustainability of concrete is to use sea water for its production. This is due to the fact that fresh water is now the most valuable commodity in the 21st century, as a result of over-exploitation of available deposits [28]. According to the data published by United Nations Organization [29], over 40% of the world’s population faces a scarcity of fresh water. At the same time, consumption of water for the production of concrete makes up 9% of the total worldwide industrial water consumption [30]. Great interest is now being taken in using sea water as an alternative solution in order to impede the exploitation of freshwater [31]. Furthermore, due to high costs for desalination, as well as a high demand for concrete production, there has been a keen interest in incorporating sea water in concrete [32].

### 1.2. The Effect of Sea Water on Performance Properties of Cement-Based Materials: The-State-Of-The-Art

Sea water can be used as an activator of the GGBFS as a result of the action of salts of strong acids, such as chlorides and sulfates, that are contained in sea water.

It was shown that sea water could promote the increase in early strength gain of the concretes based on both alkali-activated slag cement [33] and blast furnace cement [34]. The cements based on the GGBFS as a main constituent can, in most cases, reveal their high-performance properties due to a combined effect of alkali metal compounds and salts of strong acids, such as sodium sulfate [35], sodium chloride [36] and other chlorides (KCl, CaCl_2_ and MgCl_2_) [37].

However, a negative effect of sea water on the strength of the cements has also been reported. For example, the lower early strength (at 3 d) of the alkali-activated slag cement was reported in [38]. This effect was caused by a deceleration of this type of cement’s hydration process due to the formation, on the surface of the GGBFS particles, of low soluble or insoluble compounds such as brucite (Mg(OH)_2_), halite (NaCl), gypsum (CaSO_4_∙2H_2_O), etc. as a result of its interaction with the salts of strong acids. Brucite and gypsum could evidently bring some crystallization pressure into micropores resulting in the lower late strength [32].

Sea water can also change the pore structure of concrete. As was shown in [39], the alkali-activated GGBFS pastes mixed with sea water were characterized by an increased total porosity and a denser structure, resulting in a lower water absorption. However, the use of sea water can increase the open capillary porosity due to the less-densified gel microstructure as well as the transformation of large pores into pores of a smaller size due to precipitation of the mentioned hydration products as a result of interaction of the GGBFS with salts of strong acids [38]. The greater volume of open capillary pores in concrete based on alkali-activated GGBFS and mixed with sea water predetermines their lower durability due to the lower resistance to the ingress of aggressive substances [40].

The abovementioned controversial results regarding the effect of sea water on the pore structure and strength of the GGBFS containing cement materials can support an assumption about their dependence from a chemical nature, on the alkali metal compound and its content [17].

Another considerable factor affecting performance properties of the cement materials is the water content required to provide a desirable consistency, determined by the chemical structure of the plasticizing admixtures used. However, the increase in the GGBFS content and the presence of alkali metal compound leads to a diminution in the effectiveness of plasticizing admixtures [41]. The principles to be considered for the choice of suitable admixtures depending on the content of GGBFS and the alkali metal compound in cement have been proposed: plasticizing admixtures [42,43], shrinkage reducing admixtures [44,45,46] and expanding admixtures [47,48]. For example, the highest plasticizing effect in the case of concretes based on the mentioned cement can be provided thanks to sodium lignosulfonate, sodium gluconate, polyols and by other acyclic low and high molecular compounds [42].

However, the corrosion of steel reinforcement resulting from the action of chlorides and sulfates is another concern with regard to the concretes mixed with sea water [49]. The corrosion of steel reinforcement in concretes mixed with sea water can be prevented by decreasing the contents of Cl^−^-ions in a pore solution through their chemical adsorption by hydrates of cement [50]. It is also well-known that low soluble hydration products of GGBFS activated by alkali metal compounds are able to bind Cl^−^-ions, both in a physical and chemical way [51]. The alkaline aluminosilicate hydrates, being analogues of natural zeolites [17], can occlude Cl^−^- and SO_4_^2−^-ions. Moreover, Cl^−^-ions can be chemically bound by hydrotalcite ([Mg_3_Al(OH)_8_]Cl·3H_2_O) and hydrocalumite (3CaO·Al_2_O_3_·CaCl_2_·10H_2_O) [52].

The AFm phases (Al_2_O_3_-Fe_2_O_3_-mono) are characterized by a greater stability, in comparison to the AFt phases (ettringite), when increasing the alkalinity of the hydration medium [53,54]. The AFm phases can include various anions: Cl^−^, SO_4_^2−^, CO_3_^2−^, OH^−^, etc. The AFm phases can be represented by monocarboaluminate, hemicarboaluminate, stratlingite, hydroxy-AFm and monosulfoaluminate [55]. The presence of the nitrate-containing AFm phase, as well as those containing Cl^−^ and SO_4_^2−^-ions, has also been demonstrated in [56,57].

In this regard, the use of complex additives based on salts of strong acids has been proposed to minimize drying shrinkage, as well as in order to enhance crack resistance of the plasticized alkali-activated slag cement concretes, as a result of the lower water requirement, accelerated crystallization, alteration of pore structures and morphology of the hydrated phases [56].

The above results show that the use of sea water as a mixing water can lead to insufficient durability of the reinforced concrete structures due to the lower strength and deterioration of the pore structure, as well as the corrosion of steel reinforcement, under the action of Cl^−^ and SO_4_^2−^-ions. All this requires finding effective solutions to mitigate the content of the Cl^−^ and SO_4_^2−^-ions in the pore solution.

A solution is to use, as admixtures, the calcium aluminate cement and natural zeolite in order to bind the Cl^−^ and SO_4_^2−^-ions both physically and chemically and to enhance the durability of concretes mixed with sea water. Calcium aluminate cement was chosen to initiate the formation of high-calcium aluminate hydrates (3CaO∙Al_2_O_3_∙10H_2_O) due to their interaction with Portland cement clinker [58]. In its turn, 3CaO∙Al_2_O_3_∙10H_2_O ensures the binding of Cl^−^ and SO_4_^2−^-ions by the AFm phases, like 3CaO∙Al_2_O_3_∙CaCl_2_ (SO_4_)∙10H_2_O [15]. The application of natural zeolite (clinoptilolite) can also be a means to enhance the durability of concrete due to the occlusion of the Cl^−^ and SO_4_^2−^-ions. Clinoptilolite adds to the occlusion function of the alkaline aluminosilicate hydrates (analogues of natural zeolites) during hydration of the blast furnace cement.

Thus, the purpose of this research, the results of which are presented in this paper, was to combine minor additional constituents of blast furnace cement such as alkali metal compounds, calcium aluminate cement and clinoptilolite to enhance the durability of the plasticized concretes mixed with sea water due to the mitigation of corrosion initiated by the Cl^−^ and SO_4_^2−^-ions. The novelty of the study is to ensure advanced activity of blast furnace cement and to provide reliable binding of the mentioned ions during hydration while solving the problem connected with a lack of fresh water for mixing.

## 2. Materials and Methods

### 2.1. Materials

The main constituents of the blast furnace cement (CEM III/C, in accordance with EN 197-1:2011) were presented by:

1. GGBFS (PJSC “Ilyich iron and steel works”, Ukraine) with a basicity modulus of 1.11 and a glass phase content of 84% by mass;
2. Portland cement clinker (PJSC Ivano-Frankivskcement, Ukraine).

The blast furnace cement was composed of 95% of GGBFS and 5% Portland cement clinker (by mass).

Moreover, minor additional constituents (further, MACs) in blast furnace cement were taken in a quantity below 5% by the mass of main constituents and presented by:

3. An alkali metal compound (soda ash Na_2_CO_3_ (in accordance with CAS 497-19-8) or sodium metasilicate pentahydrate Na_2_SiO_3_∙5H_2_O (in accordance with CAS 10213-79-3));
4. Calcium aluminate cement ISTRA 40 (HeidelbergCement, Germany);
5. Natural zeolite (clinoptilolite) powder (JSC Zeolite-Bio, Ukraine), with a particle size <0.1 mm.

Chemical composition of dry constituents is presented in Table 1.

Two compositions of the MACs for the blast furnace cement were used, with percentage by mass of the main constituents:

6. Soda ash Na_2_CO_3_—3, calcium aluminate cement—1 and clinoptilolite—1;
7. Sodium metasilicate pentahydrate Na_2_SiO_3_∙5H_2_O—2, calcium aluminate cement—1 and clinoptilolite—1.

Two surfactants were also used: sodium lignosulphonate (in accordance with CAS 8061-51-6 and with pH ≥ 8.5) and sodium gluconate (in accordance with CAS 527-07-1). A quartz sand in accordance with the EN 196-1 was used in the fine aggregate blast furnace cement concretes (cement-to-sand ratio = 1:3).

Fresh water and artificial sea water (composed of the following salts, with percentage by mass: NaCl—78.70, MgCl_2_—9.80, MgSO_4_—5.76, CaSO_4_—3.75, KCl—1.73, CaCO_3_—0.29) were used. This oxide composition is similar to 99.90% of salts contained in natural sea water. The total concentration of the salts was 35 g/L. Rebars with a length of 120 mm and a diameter of 4.1–4.3 mm were used as the reinforcement steel.

In order to simulate effects rendered by sea water constituents (salts of strong acids), the influence of NaCl and Na_2_SO_4_ on the strength of the fine concrete based on blast furnace cement was determined. 

A number of the blast furnace cement concrete mixtures were designed. The concrete mixture compositions of the blast furnace cement concretes are presented in Table 2.

Alkali-activated slag cement based on the GGBFS was used as model to demonstrate the role played by the salts of strong acids in the presence of the mentioned alkali metal compounds.

### 2.2. Mixing Procedures

The blast furnace cement concrete mixtures were prepared in a mixer “Raimondi Iperbet” (Italy; engine: bucket rpm reduction ratio = 25:1; 1 bucket—45 L, 230V/50—60 Hz).

Then, 40 × 40 × 160 mm prisms of specimens were cast and cured under normal conditions (t = 20 ± 2 °C, R.H. = 95 ± 5%). The values of the compressive strength of six specimens, with a mean value taken as representative, were determined.

### 2.3. Testing Methods

The consistency (workability) of fresh concrete was determined by a cone slump, in accordance with the EN 12350-2 standard. The monitoring of structure formation of the blast furnace cement was carried out by X-ray diffraction (XRD), using a single-crystal X-ray diffractometer with a copper anode “DRON-3M” (Russia) with CuKα radiation (λ = 0.1542 nm at 40 kV and 25 mA), differential-thermal analysis (DTA) with a MOM derivatograph system (F. Paulik, J. Paulik and L. Erdey; MOM, Budapest; a temperature interval of 20–1000 °C at a heating rate of 10 °C/min) and an electronic microscope with a microanalyzer (REMMA 102-02, SELMI, Ukraine). The settings of the scanning electron microscope were as follows: accelerating voltage—up to 35 kV; SEI mode resolution—5 nm; magnification—from 10 to 250,000. The settings of the energy dispersive X-ray spectrometer were as follows: analyzed element range—from Na; energy resolution—143 eV at MnKα; energy range—up to 30 kV).

The water absorption and porosity of the blast furnace cement concrete were measured in accordance with the national standard of Ukraine, DSTU B V.2.7-170:2008. The concrete cubes (100 mm) after 28 days of hardening (t = 20 ± 2 °C, R.H. = 95 ± 5%) were dried up to a constant weight mass at 105 ± 10 °C. Then, the specimens were saturated with water until a constant weight was obtained at t = 20 ± 2 °C. The values of porosity were calculated from the values of the average density and water absorption.

The ultrasonic pulse velocity of the blast furnace cement concrete was tested in accordance with the national standard of Ukraine, DSTU B V.2.7-226:2009. The settings of the ultrasonic pulse velocity tester UKB-1M were indirect and direct transmission, longitudinal and surface waves, and a working frequency of 100 kHz.

The state of the embedded steel rebars in the plasticized blast furnace cement concretes mixed with sea water were estimated according to the following method: the basic rebars, with a length of 120 ± 2 mm and a diameter between 3 mm and 6 mm, were embedded in 40 × 40 × 160 mm blast furnace cement concrete specimens. The rebars were degreased with acetone and weighed, to an accuracy of ± 0.001 g, before embedding. After the specimens hardened under normal conditions (t = 20 ± 2 °C, R.H. = 95 ± 5%), the basic bars were removed from the blast furnace cement concretes and etched for 25 ± 5 min in 10% hydrochloric acid solution, with an addition of urotropine (1% by mass of the acid) to remove any remaining cement stone and corrosion products. The reference rebars, which were not embedded in the concrete, were weighed and etched simultaneously with the basic rebars. After etching, the basic and reference rebars were cleaned with distilled water and immersed in a fat solution of sodium nitrate for 5 min. The rebars were then wiped with filter paper, dried and weighed. The mean mass loss of the basic and reference rebars was calculated as the ratio of the mean differences of the mass of the rebars, before and after etching, to the surface area. The mass loss was calculated as a difference between the mean loss of the basic and reference rebars.

## 3. Results and Discussion

### 3.1. Activation of the Ground Granulated Blast Furnace Slag by the Salts of Strong Acids in Combination with Alkali Metal Compounds

**Figure 1:**
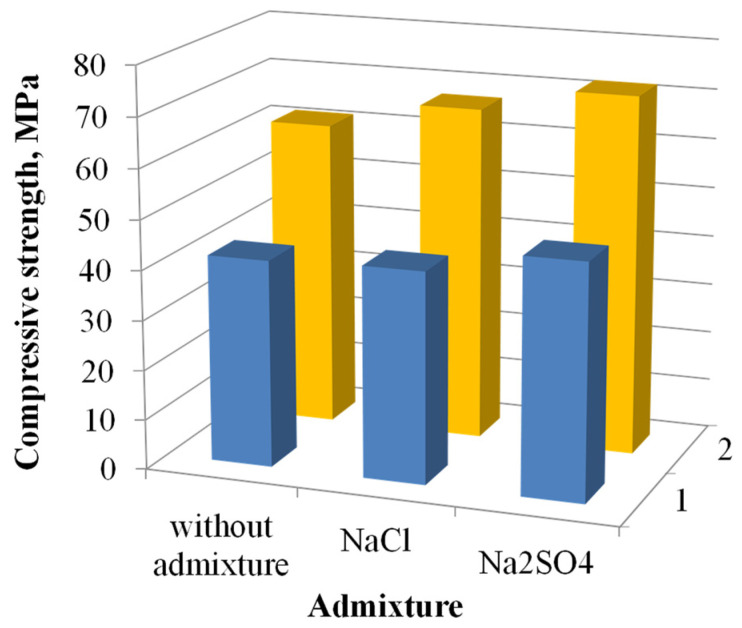
The 28-day compressive strength of the alkali-activated slag cement concretes mixed with fresh water: 1—based on Na_2_CO_3_; 2—based on Na_2_SiO_3_∙5H_2_O.

A comparative analysis showed that salts of alkaline metals, which can provide an alkaline reaction, activated the GGBFS more strongly than hydroxides of alkaline metals. Furthermore, the period of the structure and properties formation of the alkali-activated (Na_2_SiO_3_∙5H_2_O) slag cement concrete was shorter than that of the alkali-activated (Na_2_CO_3_) slag cement concrete (Figure 1). The experimental data error was in compliance with the permissible limits (no more than 5%).

A combined application of the salts of strong acids with Na_2_CO_3_ or Na_2_SiO_3_∙5H_2_O predetermines early strength development of alkali-activated slag cement concrete. The effects of sea water on the performance properties of the alkali-activated slag cement concretes were thus simulated. However, the effectiveness of the salts of strong acids was defined by anions. For example, the use of NaCl for modification of the alkali-activated (Na_2_CO_3_) slag cement concrete did not affect the standard strength (28 d). Furthermore, the use of Na_2_SO_4_ resulted in a 13% strength increase, compared to the alkali-activated slag cement concrete without admixture. In the case of the alkali-activated (Na_2_SiO_3_∙5H_2_O) slag cement concrete concretes, the use of the salts of strong acids (NaCl and Na_2_SO_4_) ensured a strength increase by 9% and 17%, respectively.

The results mentioned above confirm a role played by the salts of strong acids of sea water in the additional activation of the GGBFS. As such, the GGBFC in the presence of alkali metal compounds is characterized by the increased content of a gel, as a result of the release of the anions ≡ Si–O– from a polycondensation reaction and their transfer into a colloid phase during hydration. It can be predicted that the interaction of the anions ≡ Si–O– with the cations Cl^−^ and SO_4_^2−^, side by side with the formation of aquocomplexes Si–O–Cl–OH and Si–O–SO_3_–OH, can ensure the enhanced binding of aggressive ions by the hydration products of GGBFC-activated alkali metal compounds [17].

### 3.2. Structure Formation of the Blast Furnace Cement Mixed with Sea Water

The effects of the alkali metal compound (Na_2_CO_3_ or Na_2_SiO_3_∙5H_2_O) alone as well as in combination with calcium aluminate cement as MACs on the structure formation of the blast furnace cement mixed with sea water are shown below.

#### 3.2.1. Effect of Na_2_CO_3_ as a Sole Minor Additional Constituent

**Figure 2:**
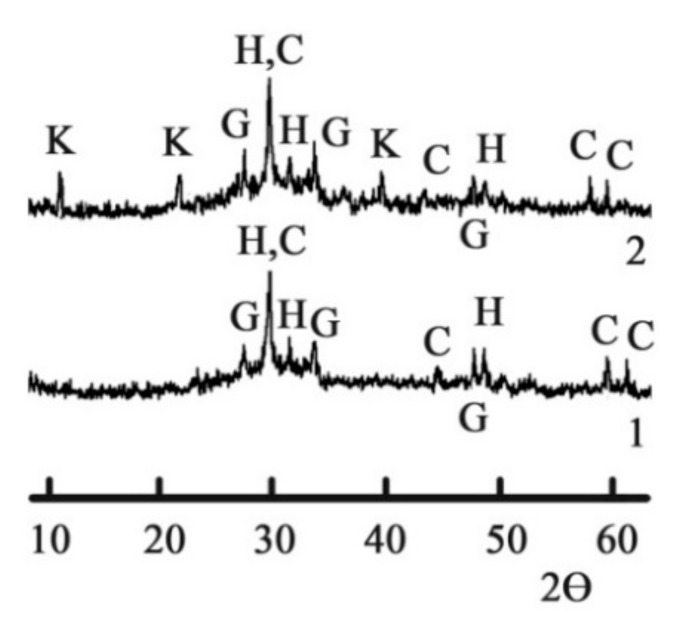
The XRD of the 180-day hydrated blast furnace cement mixed with sea water and containing, as minor additional constituents, 1—Na_2_CO_3_ and 2—Na_2_CO_3_ in combination with calcium aluminate cement. Legend: H—calcium silicate hydrates CSH (B), G—gyrolite, C—calcite, K—Kuzel’s salt.

The XRD allowed us to identify slightly crystallized low-calcium silicate hydrates, such as CSH (B) (d = 0.307; 0.280; 0.183 nm) and gyrolite 2CaO∙3SiO_2_∙2H_2_O (d = 0.33; 0.268; 0.180 nm) after 180 d of hydration (Figure 2, curve 1). Calcite (d = 0.307; 0.191; 0.160; 0.152 nm) was identified as well. These hydrates are typical for GGBFS activated by Na_2_CO_3_ [59].

Chlorine- and sulfate-binding zeolite-like minerals, which are similar to nosean Na_8_ (Al_6_Si_6_O_24_)(SO_4_)·H_2_O, sodalite Na_4_(Si_3_A_l3_)O_12_Cl, cancrinite (Na, Ca)_8_(Al_6_Si_6_O_24_)(CO_2_,SO_4_)_2_·2H_2_O and others, can also be predicted to appear [17,60]. However, these hydrates were not identified because of their submicrocrystalline state.

**Figure 3:**
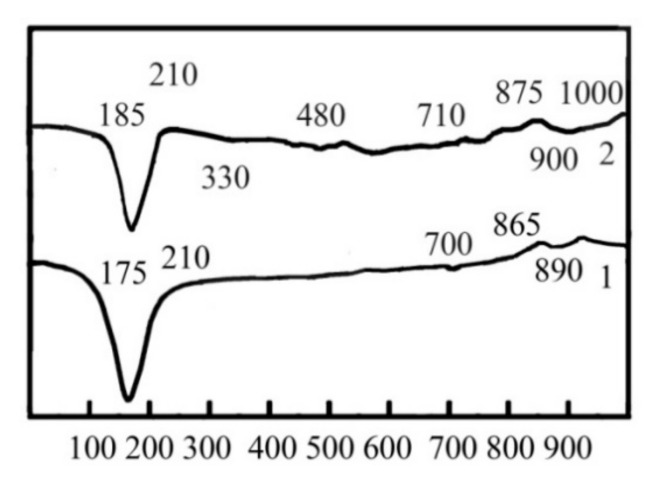
DTA results of the 180-day hydrated blast furnace cement mixed with sea water and containing, as minor additional constituents, 1—Na_2_CO_3_ and 2—Na_2_CO_3_ in combination with calcium aluminate cement.

DTA confirmed the formation of slightly crystallized calcium silicate hydrates CSH(B) via the endothermic effect at 175 °C (dehydration) and the exothermic effect at 865 °C (recrystallization into wollastonite). The endothermic effects at 175 °C and 700 °C (stepped dehydration) and the exothermic effect at 865 °C (recrystallization into wollastonite) are typical for gyrolite 2CaO∙3SiO_2_∙2H_2_O (Figure 3, curve 1). The endothermic effect at 890 °C allowed us to confirm the presence of CaCO_3_.

**Figure 4:**
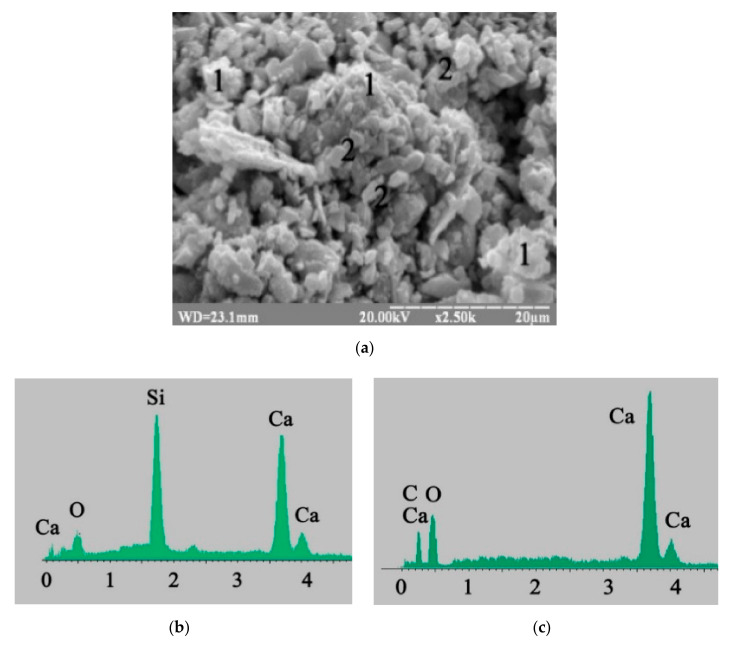
Microstructure of the 180-day hydrated blast furnace cement mixed with sea water and containing Na_2_CO_3_ as minor additional constituents: (**a**) SEM image; (**b**) Microprobe analysis in point 1; (**c**) Microprobe analysis in point 2.

According to electron microscopy (Figure 4a), gel-like low-calcium silicate hydrates CSH (B) (percentage of oxide content by mass in a probe: CaO—30.19 and SiO_2_—35.42) (Figure 4b) and prismatic calcite CaCO_3_ (percentage of oxide content by mass in a probe: CaO—53.78 and CO_2_—41.29) (Figure 4c) were identified in blast furnace cement containing Na_2_CO_3_.

#### 3.2.2. Effect of Na_2_CO_3_ and Calcium Aluminate Cement as Minor Additional Constituents

The chloride–sulfate AFm phases (Kuzel’s salt), 3CaO∙Al_2_O_3_∙0.5CaCl_2_∙0.5SO_4_∙10H_2_O (d = 0.83; 0.42; 0.23 nm) [61], were also identified in the hydration products of the blast furnace cement containing Na_2_CO_3_ and calcium aluminate cement as MACs (Figure 2, curve 2). These phases were formed as a result of chemical binding of the Cl^−^ and SO_4_^2−^-ions by calcium aluminate hydrate 3CaO∙Al_2_O_3_∙10H_2_O. Calcium aluminate hydrate in its turn was formed due to the interaction between the hydration products of Portland cement clinker and calcium aluminate cement.

The presence of Kuzel’s salt in the hydration products of the blast furnace cement containing Na_2_CO_3_ and calcium aluminate cement was confirmed by the endothermic effects at 330 °C (dehydration) and 480 °C (removal of Cl^−^), as well as by the exothermic effect at 1000 °C (decomposition of sulfate) (Figure 3, curve 2). The relocation of these effects to the field of higher temperatures ensured the formation of CSH (B) and gyrolite with the higher degree of crystallization.

**Figure 5:**
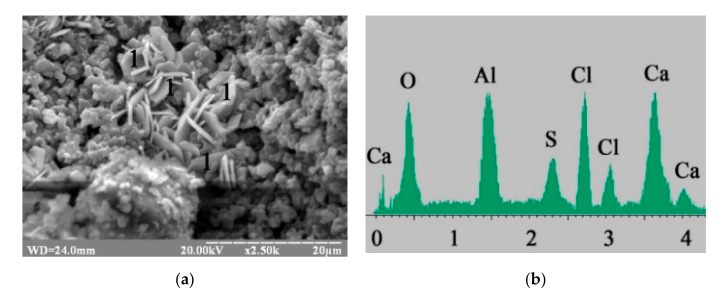
Microstructure of the 180-day hydrated blast furnace cement mixed with sea water and containing Na_2_CO_3_ in combination with calcium aluminate cement as minor additional constituents: (**a)** SEM image; (**b**) Microprobe analysis in point 1.

The application of Na_2_CO_3_ in combination with calcium aluminate cement in the blast furnace cement resulted in the formation of low-calcium silicate hydrates with the higher level of crystallization, as well as thin, hexagonal plates of Kuzel’s salt (Figure 5a). The contents of the oxides confirmed the formation of Kuzel’s salt (percentage by mass: CaO—32.72, Al_2_O_3_—21.51, Cl—10.27, SO_3_—9.56) (Figure 5b).

#### 3.2.3. Effect of Na_2_SiO_3_∙5H_2_O as a Sole Minor Additional Constituent

**Figure 6:**
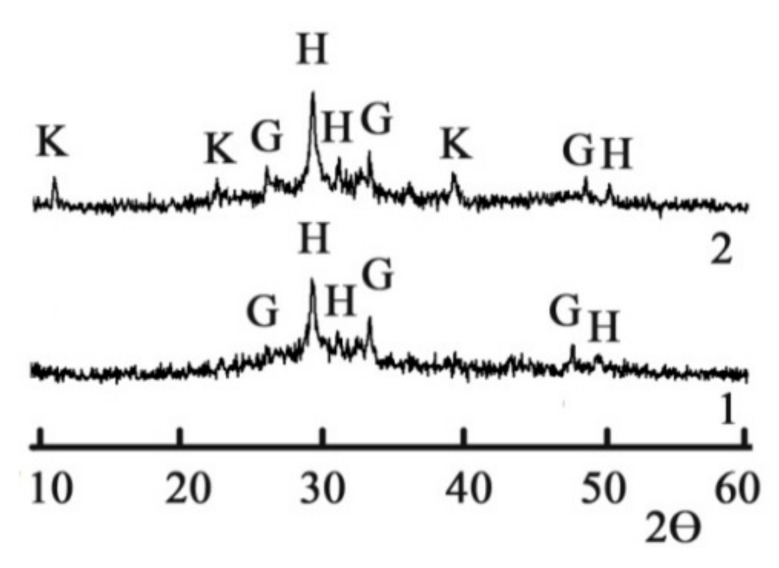
The XRD of the 180-day hydrated blast furnace cement mixed with sea water and containing 1—Na_2_SiO_3_∙5H_2_O and 2—Na_2_SiO_3_∙5H_2_O in combination with calcium aluminate cement. Legend: H—calcium silicate hydrates CSH (II), G—gyrolite, K—Kuzel’s salt.

The results of XRD showed the formation of slightly crystallized low-calcium silicate hydrates, such as CSH (B) and gyrolite (Figure 6, curve 1). The presence of the zeolite-like minerals (similar to nosean, sodalite, cancrinite, etc. in terms of composition), which can occlude Cl^−^ and SO_4_^2−^-ions, could be assumed.

**Figure 7:**
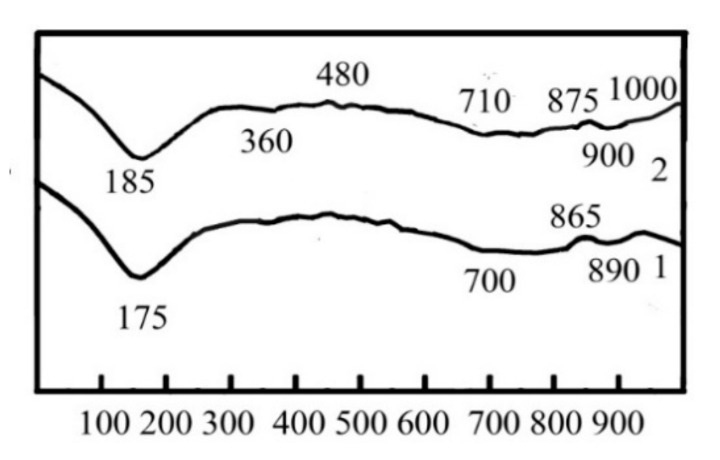
DTA results of the 180-day hydrated blast furnace cement mixed with sea water and containing 1—Na_2_SiO_3_∙5H_2_O and 2—Na_2_SiO_3_∙5H_2_O in combination with calcium aluminate cement.

**Figure 8:**
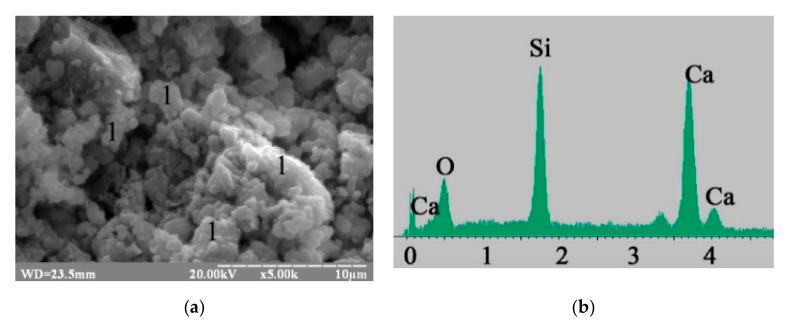
Microstructure of the 180-day hydrated blast furnace cement mixed with sea water and containing Na_2_SiO_3_∙5H_2_O: (**a**) SEM image; (**b**) Microprobe analysis in point 1.

The presence of the silicate hydrates mentioned above was confirmed (Figure 7, curve 1). Gel-like low-calcium silicate hydrates CSH (B) (percentage content of oxides by mass in the probe: CaO—32.35; SiO_2_—34.71) were identified in blast furnace cement containing Na_2_SiO_3_∙5H_2_O (Figure 8).

#### 3.2.4. Effect of Na_2_SiO_3_∙5H_2_O and Calcium Aluminate Cement as Minor Additional Constituents

According to XRD, low-calcium silicate hydrates and Kuzel’s salt were identified as the hydration products of the blast furnace cement containing Na_2_SiO_3_∙5H_2_O in combination with calcium aluminate cement (Figure 6, curve 2). The presence of these hydrates was supported by the date of DTA (Figure 7, curve 2). The displacement of these effects to the field of the higher temperatures ensured the formation of CSH (B) and gyrolite with the higher degree of crystallization.

**Figure 9:**
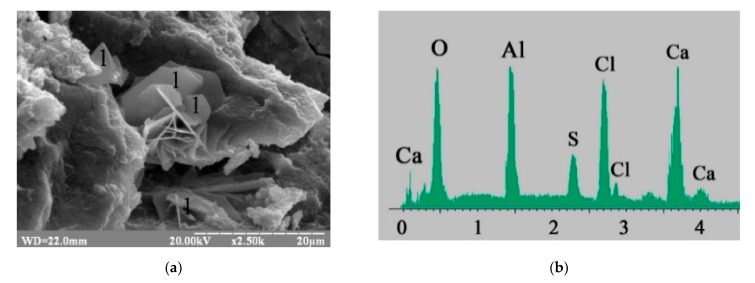
Microstructure of the 180-day hydrated blast furnace cement mixed with sea water and containing Na_2_SiO_3_∙5H_2_O in combination with calcium aluminate cement: (**a**) SEM image; (**b**) Microprobe analysis in point 1.

A phase composition of the blast furnace cement containing Na_2_SiO_3_∙5H_2_O in combination with calcium aluminate cement (Figure 9) was presented by the low-calcium silicate hydrates as well as by the thin hexagonal plates of Kuzel’s salt (contents of oxides by mass in the probe: CaO—31.22, Al_2_O_3_—24.87, Cl—12.43, SO_3_—10.94).

Thus, it is clear that calcium aluminate cement as a MAC initiates chemical binding of the Cl^−^ and SO_4_^2−^-ions in the structure of the hydrated blast furnace cement mixed with sea water. The interaction between the hydration products of Portland cement clinker and calcium aluminate cement resulted in the formation of high-calcium aluminate hydrates like 3CaO∙Al_2_O_3_∙10H_2_O, with chemical binding of the aggressive ions into Kuzel’s salt.

The above-mentioned structure formation of the blast furnace cement mixed with sea water showed the efficiency of the alkali metal compound (Na_2_CO_3_ or Na_2_SiO_3_∙5H_2_O) in combination with calcium aluminate cement as MACs in enhancing the durability of the concretes. It was determined that alkali metal compounds activate hydraulic properties of the GGBFC. In its turn, calcium aluminate cement initiates the mitigation of free Cl^−^ and SO_4_^2−^-ions in the pore solution of the cement stone due to chemical binding into Kuzel’s salt.

### 3.3. Performance Properties of the Plasticized Concretes Based on Blast Furnace Cement Containing an Alkali Metal Compound in Combination with Calcium Aluminate Cement and Clinoptilolite as Minor Additional Constituents

#### 3.3.1. Consistency

A consistency class of S4 (slump 160 mm–210 mm), conforming to EN 12350-2, was reported for the blast furnace cement concretes. The slump values of the concrete mixtures were 170 mm and 180 mm, with a W/C ratio of 0.44 and 0.41, when mixing with sea water and in cases where soda ash and sodium metasilicate were the alkali metal compounds. The fresh concretes based on the blast furnace cement mixed with fresh water were obtained with the slump values of 180 mm and 200 mm, with W/C ratios of 0.46 and 0.43, respectively. As it follows, consistency of the fresh blast furnace cement concretes could be adjusted by the use of sea water.

#### 3.3.2. Compressive Strength

**Figure 10:**
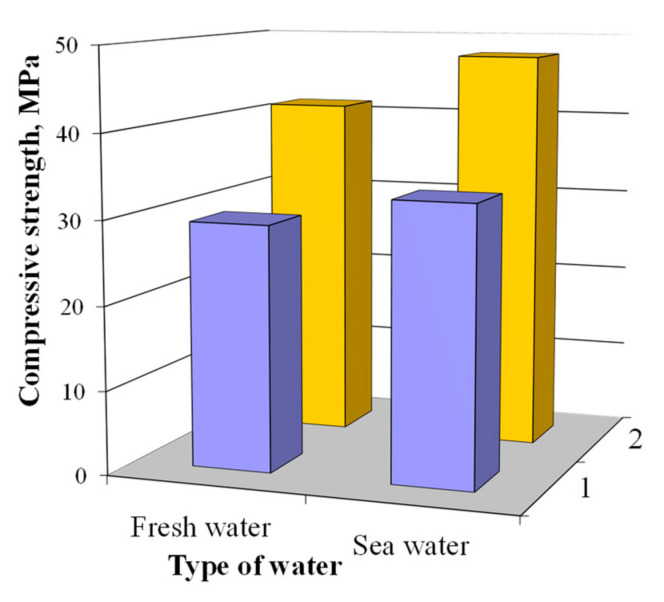
The 28-day compressive strength of the plasticized concrete based on blast furnace cement containing calcium aluminate cement and clinoptilolite in combination with 1—Na_2_CO_3_ and 2—Na_2_SiO_3_∙5H_2_O.

The plasticized blast furnace cement concretes, not depending upon alkali metal compounds, mixed with sea water exhibited higher compressive strength values compared to those of the concretes mixed with fresh water (Figure 10).

The compressive strength of the concrete based on blast furnace cement containing Na_2_CO_3_ in combination with calcium aluminate cement and clinoptilolite as MACs and mixed with sea water was 35.7 MPa and 21.8% higher than that of the specimen mixed with fresh water (29.3 MPa). The compressive strength of the concrete based on blast furnace cement containing Na_2_SiO_3_∙5H_2_O in combination with calcium aluminate cement and clinoptilolite as MACs and mixed with sea water was by 10.9% higher than that of the analogue mixed with fresh water (45.8 and 41.3 MPa, respectively).

The absence of strength losses due to the crystallization pressure of gypsum CaSO_4_∙2H_2_O as a result of interaction of the GGBFS with salts of strong acids can be attributed to the chemical binding of SO_4_^2−^-ions by the AFm phases, as well as to the occlusion of these ions by clinoptilolite [37].

#### 3.3.3. Durability

The durability of concrete is determined by both the strength and density of the structure. The latter property can be characterized by the values of porosity, ultrasonic pulse velocity, as well as water absorption. The blast furnace cement concrete mixed with sea water exhibited a different pore structure than the one mixed with fresh water (Table 3).

**Figure 11:**
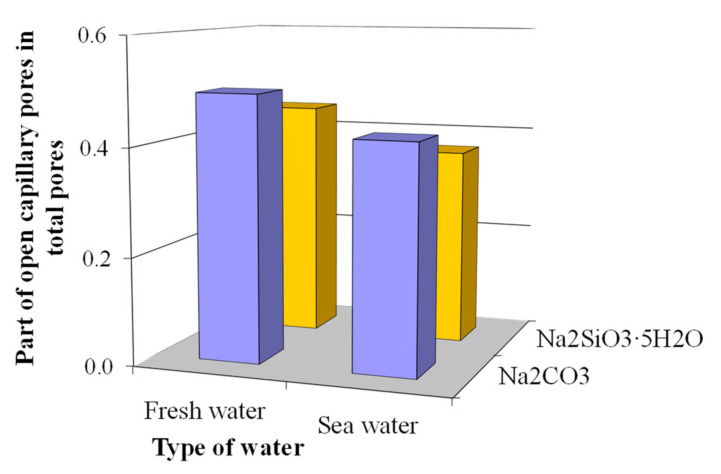
Specific volume of open capillary pores in the plasticized concrete based on blast furnace cement.

**Figure 12:**
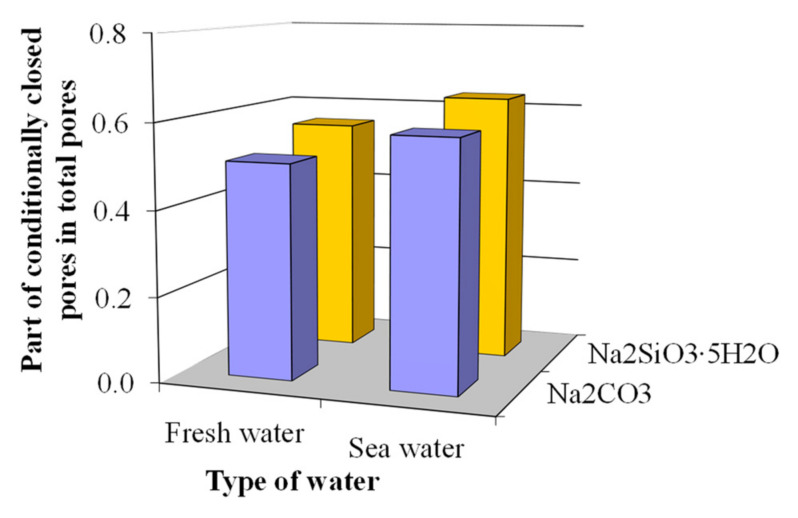
Specific volume of conditionally closed pores in the plasticized concrete based on blast furnace cement.

In the case of sea water in the concrete based on blast furnace cement containing Na_2_CO_3_ in combination with calcium aluminate cement and clinoptilolite as MACs, the part of the open capillary pores in total porosity decreased from 0.49 down to 0.42 (Figure 11) and increased the part of conditionally closed pores from 0.51 up to 0.58 (Figure 12) compared to an analogue mixed with fresh water. The application of sea water in the concrete based on the blast furnace cement containing Na_2_SiO_3_∙5H_2_O in combination with calcium aluminate cement and clinoptilolite as MACs allowed the share of open capillary pores to decrease from 0.44 down to 0.37 (Figure 11) and the share of conditionally closed pores to increase from 0.56 up to 0.63 (Figure 12).

The total porosity of the concretes based on blast furnace cement containing an alkali metal compound (Na_2_CO_3_ or Na_2_SiO_3_∙5H_2_O) in combination with calcium aluminate cement and clinoptilolite as MACs and mixed with sea water was by 5.1 and 4.7% higher, accordingly, compared to that in case of fresh water (Table 3).

Thus, the concrete based on blast furnace cement containing an alkali metal compound (Na_2_CO_3_ or Na_2_SiO_3_∙5H_2_O) in combination with calcium aluminate cement and clinoptilolite as MACs and mixed with sea water was characterized by a more perfect pore structure due to the filling of the pore space by Kuzel’s salt as a result of chemical binding of the Cl^−^ and SO_4_^2−^-ions.

**Figure 13:**
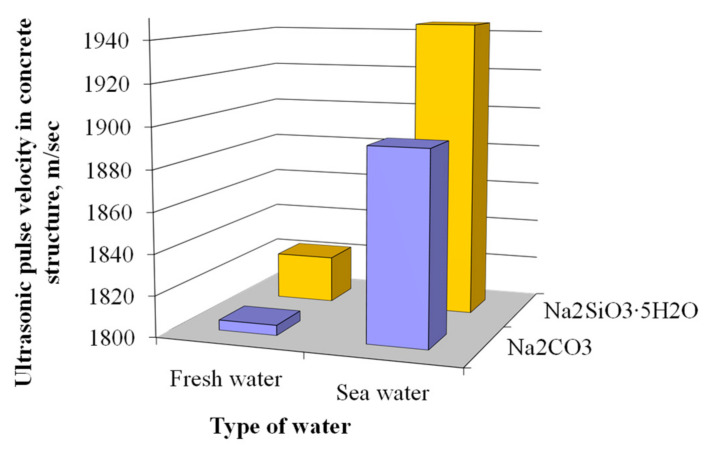
Ultrasonic pulse velocity in the plasticized concrete based on blast furnace cement.

The specified changes in the pore structure result in the denser structure of the plasticized blast furnace cement concrete mixed with sea water. The ultrasonic pulse velocity measured in the structure of the concretes mixed with sea water was by 4.9 and 6.7% higher than those of the analogues mixed with fresh water (Figure 13).

**Figure 14:**
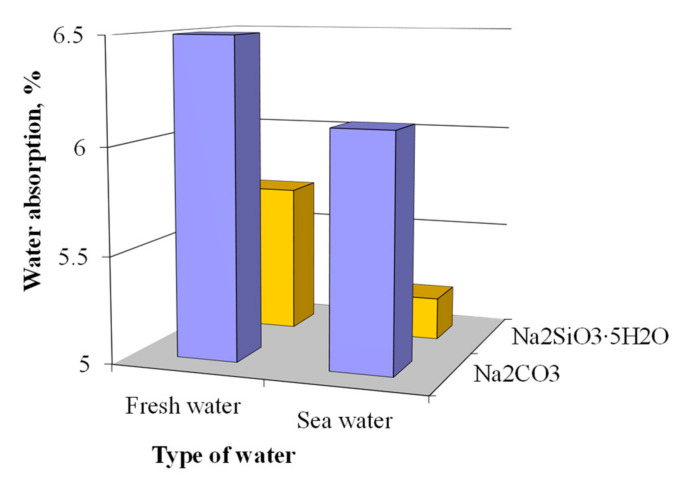
Water absorption of the plasticized concrete based on blast furnace cement.

The higher density of the blast furnace cement concretes mixed with sea water resulted in the decrease of their water absorption. The water absorption of the concrete containing an alkali metal compound (Na_2_CO_3_ or Na_2_SiO_3_∙5H_2_O) in combination with calcium aluminate cement and clinoptilolite as MACs and mixed with sea water was 6.2% and 8.8% lower, respectively, compared to those of the analogues mixed with fresh water (Figure 14).

Thus, the application of an alkali metal compound (Na_2_CO_3_ or Na_2_SiO_3_∙5H_2_O) in combination with calcium aluminate cement and clinoptilolite as MACs of the blast furnace cement resulted in the higher density of the plasticized concrete mixed with sea water.

#### 3.3.4. State of Steel Reinforcement

**Figure 15:**
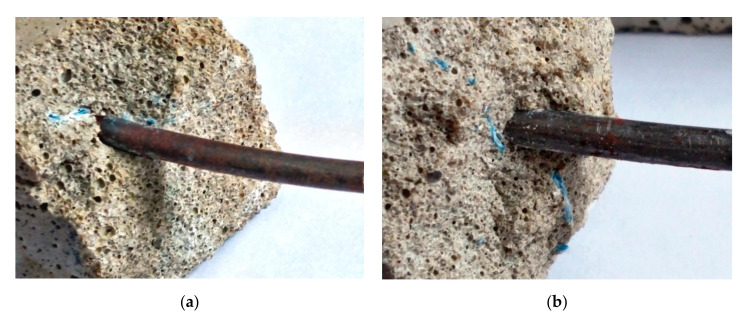
Steel rebars embedded in the plasticized concretes based on blast furnace cement containing Na_2_CO_3_ (**a**) or Na_2_SiO_3_∙5H_2_O (**b**) in combination with calcium aluminate cement and clinoptilolite as minor additional constituents and mixed with sea water after 180 days of hardening (Magnification: ×4).

The effects of MACs of blast furnace cement on the protective properties of the plasticized concrete concerning steel reinforcement were also investigated (Figure 15). The mass loss of steel rebars, which were extracted from the plasticized blast furnace cement concrete that was fixed after 180 days of hardening, were measured (Table 4).

The values of mass loss were in compliance with mandatory requirements (no more than 10 g/m^2^), in accordance with the DSTU B V.2.6-181 standard, and showed good protective properties of the plasticized concrete based on blast furnace cement for steel reinforcement.

The blast furnace cement containing Na_2_SiO_3_∙5H_2_O as a MAC ensured formation of a denser concrete with advanced protective properties for steel reinforcement if compared with an analogue containing Na_2_CO_3_. Additional enhancement of the plasticized concrete based on blast furnace cement performance properties could be provided by an alkali metal compound in combination with calcium aluminate cement and clinoptilolite as MACs. As such, in case of the concrete based on blast furnace cement containing Na_2_CO_3_ in combination with calcium aluminate cement and clinoptilolite as MACs, the application of one resulted in a 3.5-times lower mass loss than the value measured in concrete based on the blast furnace cement containing Na_2_CO_3_ alone. For the concrete based on blast furnace cement containing Na_2_SiO_3_∙5H_2_O, the addition of calcium aluminate cement and clinoptilolite as MACs resulted in a 3.1-times lower mass loss.

## 4. Conclusions

1. The proposed combination of the alkali metal compounds, calcium aluminate cement and clinoptilolite as minor additional constituents of the blast furnace cement is a way to allow enhanced durability of the plasticized concretes mixed with sea water. The alkali metal compounds promote an increased strength gain due to activation of the ground granulated blast furnace slag. The calcium aluminate cement promotes the advanced chemical binding of the Cl^−^ and SO_4_^2−^-ions into Kuzel’s salt, whereas the use of clinoptilolite ensures the occlusion of these ions.
2. The additional effect on the activation of the ground granulated blast furnace slag as the main constituent of blast furnace cement is provided by the salts of strong acids in combination with the alkali metal compounds, which provide an alkaline reaction in water.
3. The proposed composition of the minor additional constituents ensures the lower open capillary porosity (by 19.3% to 21.2%) and the higher conditionally closed porosity (by 8.5% to 8.8%). This is accompanied by the lower water absorption (by 6.8% to 8.8%) of the plasticized blast furnace cement concretes mixed with sea water.
4. The binding of the Cl^−^ and SO_4_^2−^-ions was confirmed by the lower weight losses of the embedded steel rebars (by 3.1- to 3.5-times) and the higher structural density (by 4.9- to 6.7-times) as well as the higher strength of plasticized blast furnace cement concrete mixed with sea water (by 12.3- to 15.7-times) in the presence of the mentioned minor additional constituents.

## Figures and Tables

**Table 1:** Chemical composition of dry constituents (% by mass).

Material	CaO	SiO_2_	Al_2_O_3_	Fe_2_O_3_	MgO	SO_3_	TiO_2_	P_2_O_5_	K_2_O + Na_2_O
GGBFS	47.30	39.00	5.90	0.30	5.82	1.50	0.31	-	5.03
Clinoptilolite	2.10	72.50	13.10	0.90	1.07	-	0.20	0.003	-
Portland cement clinker	64.68	21.44	5.22	4.84	0.55	2.32	-	-	0.95
Calcium aluminate cement	37.15	4.78	41.00	14.05	<1.50	<0.30	-	-	-

**Table 2:** The blast furnace cement concrete mix design (% by mass).

Mix	GGBFS	Portland Cement Clinker	Na_2_CO_3_ in Combination with Calcium Aluminate Cement and Clinoptilolite	Na_2_SiO_3_∙5H_2_O in Combination with Calcium Aluminate Cement and Clinoptilolite	Plasticizing Admixture	Sand	Fresh Water	Sea Water
Mix1	21.34	1.12	-	-	0.27	67.39	9.88	-
Mix2	21.44	1.13	-	-	0.27	67.69	-	9.47
Mix3	21.01	1.11	1.11	-	0.27	66.34	10.16	-
Mix4	21.20	1.12	-	0.89	0.27	66.93	9.59	-
Mix5	21.10	1.11	1.11	-	0.27	66.64	-	9.77
Mix6	21.29	1.12	-	0.90	0.27	67.23	-	9.19

**Table 3:** Pore structure of the plasticized blast furnace cement concretes.

Mineral Materials as MACs of the Blast Furnace Cement	Type of Concrete	Open Capillary Porosity	Conditionally Closed Porosity	Total Porosity
Na_2_CO_3_ in combination with calcium aluminate cement and clinoptilolite	mixed with fresh water	14.5	14.8	29.3
	mixed with sea water	11.7	16.1	27.8
Na_2_SiO_3_∙5H_2_O in combination with calcium aluminate cement and clinoptilolite	mixed with fresh water	11.3	14.1	25.4
	mixed with sea water	8.9	15.3	24.2

**Table 4:** The state of the steel rebars embedded in the plasticized blast furnace cement concretes mixed with sea water.

Mineral Materials as Minor Additional Constituents of Blast Furnace Cement	Mass Loss [g/m^2^]
Na_2_CO_3_	3.87
Na_2_SiO_3_∙5H_2_O	2.93
Na_2_CO_3_ in combination with calcium aluminate cement and clinoptilolite	1.12
Na_2_SiO_3_∙5H_2_O in combination with calcium aluminate cement and clinoptilolite	0.95

## Data Availability

The data are not publicly available due to works are carried out within the framework of the mentioned project, its results will be available on request after registration of report in State scientific institution Ukrainian Institute of Scientific and Technical Expertise and Information (http://www.uintei.kiev.ua/en) using the system https://nddkr.ukrintei.ua/.
